# Bacterial Pathogenesis and Antimicrobial Strategy

**DOI:** 10.3390/antibiotics12121750

**Published:** 2023-12-18

**Authors:** Juan C. Vázquez-Ucha, Marta Martínez-Guitián

**Affiliations:** 1 Servicio de Microbiología, Instituto de Investigación Biomédica A Coruña (INIBIC), Complexo Hospitalario Universitario A Coruña, Universidade da Coruña (UDC), 15006 A Coruña, Spain; 2 CIBER de Enfermedades Infecciosas (CIBERINFEC), Instituto de Salud Carlos III, 28029 Madrid, Spain; 3 NANOBIOFAR, Centre for Research in Molecular Medicine and Chronic Diseases, Universidad de Santiago de Compostela, 15782 Santiago de Compostela, Spain

Antimicrobial resistance and multidrug resistance are major global health concerns. In 2019, the number of deaths from antimicrobial resistance-related causes was estimated to have reached 5 million globally, and this number is expected to rise over the coming years [1]. In 2017, the World Health Organization (WHO) published a list of priority pathogens for which new antimicrobials are required. The pathogens *Acinetobacter baumannii* and *Pseudomonas aeruginosa* resistant to carbapenems, as well as the *Enterobacteriaceae* family resistant to carbapenems or third-generation cephalosporins, were placed in the category of greatest urgency, i.e., “critical priority”. Other microorganisms that seriously affect human health, such as *Enterococcus faecium*, *Staphylococcus aureus* and members of the genus *Salmonellae* resistant to different groups of antibiotics, were included in the second category of urgency, i.e., “high priority” [2].

Several public and private initiatives are being undertaken to develop new therapeutic agents against infections caused by multidrug-resistant pathogens. Regarding the β-lactam antibiotics, the most diverse and widely used class of antibiotics, the recently approved cefiderocol, a cephalosporin siderophore, has shown high activity against *A. baumannii*, *P. aeruginosa* and *Enterobacteriaceae* [3]. Several β-lactam/β-lactamase inhibitor combinations, such as imipenem/relebactam, meropenem/vaborbactam and sulbactam/durlobactam, have also been developed to act against these pathogens [4]. Antimicrobials belonging to other antibiotic classes have also recently been approved, and most of these are mainly active against Gram-positive bacteria. This is the case for the glycopeptides dalvabancin and oritavancin, the fluoroquinolones delafloxacin and zabofloxacin and the tetracyclines eravacycline and omadacycline, among others [5]. New antimicrobial molecules adapted from existing classical antimicrobial classes are currently at different stages of clinical development, such as the β-lactams sulopenem and benapenem, the macrolide nafithromycin and the aminoglycoside EBL-1003, among others. Hundreds of molecules are also in pre-clinical stages of development. Immunomodulatory agents and non-traditional antimicrobials, including antibodies, bacteriophages and phage-derived enzymes, are also currently at different stages of development [6].

In addition to the development of new antimicrobials, it is of vital importance to maintain the effectiveness of currently available antibiotics. This requires in-depth knowledge of the antimicrobial resistance mechanisms used by the strains causing the infection. Whole genome sequencing (WGS), increasingly used in microbiology services, can reveal the potential mechanisms of antimicrobial resistance and other relevant aspects, such as the virulence mechanisms of the strains, and can also be used to identify high-risk sequence types (STs) [7].

This editorial refers to the Special Issue entitled “Bacterial Pathogenesis and Antimicrobial Strategy”. This Special Issue highlights the importance of (i) the development and evaluation of new antimicrobial and antivirulence agents; (ii) the characterisation of new mechanisms of antimicrobial resistance; and (iii) the identification and description of multidrug-resistant strains and STs. Seven manuscripts were submitted for consideration for the Special Issue and five of them were finally accepted for publication. The contributions are listed below.

In the first contribution, Alonso-García et al. describe the importance of the β-lactamase enzyme OXA-10 (a non-carbapenemase β-lactamase) in conferring resistance to carbapenems when its expression is associated with a deficiency in *E. coli* porins. The authors proceed from the isolation and identification of two non-carbapenemase-producing carbapenem-resistant strains of *E. coli* ST57. Using WGS, cloning experiments, biochemical studies, molecular modelling and hydrolysis tests, they demonstrate the importance of OXA-10 in conferring resistance to carbapenems when its expression is combined with low bacterial permeability.

In the second contribution, Bonnin et al. evaluate the in vitro activity of the new β-lactam/β-lactamase inhibitor combinations imipenem-relebactam, meropenem-vaborbactam and ceftazidime-avibactam against 284 non-carbapenemase-producing carbapenem-resistant Enterobacterales strains. The authors demonstrate, by reference to microdilution and WGS, that the new combinations ceftazidime–avibactam, imipenem–relebactam and, to a lesser extent, meropenem–vaborbactam show very good activity (susceptibility greater than 80%) against these Enterobacterales.

In the third contribution, Elfaky et al. evaluate the ability of the α-adrenoreceptor antagonist prazosin to decrease the virulence of *Salmonella enterica* serovar Typhimurium. Based on microdilution, gene expression, antibiofilm activity, intracellular bacterial replication and in vivo antivirulence activity studies, the authors demonstrate that prazosin displays good activity against *S. Typhimurium* by decreasing the expression of key virulence factors as well as biofilm formation, allowing for increased in vivo survival of mice treated with this drug.

In the fourth contribution, Cherubini et al. analyse the genomes of 43 *A. baumannii* strains causing bloodstream infections in patients with SARS-CoV-2 co-infection. The authors use reference microdilution and WGS to study the antimicrobial susceptibility of the collection analysed, as well as the main mechanisms of antimicrobial resistance and virulence factors.

In the fifth contribution, Méndez-Moreno et al. characterise diarrheagenic *E. coli* strains isolated from healthy donors. Using microdilution, genetic analysis, adherence and biofilm formation assays, the authors show that 46 of the total 90 strains tested carry several genes involved in virulence and have an increased ability to form biofilm.

In conclusion, the manuscripts included in this Special Issue provide new information that contributes to a better understanding of the activity of novel antimicrobials, the molecular mechanisms involved in antibiotic resistance, the activity of new antivirulence agents and also the characterisation of important pathogenic strains.
